# Are Particulate Matter Exposures Associated with Risk of Type 2 Diabetes?

**DOI:** 10.1289/ehp.1002344

**Published:** 2010-11-30

**Authors:** Robin C. Puett, Jaime E. Hart, Joel Schwartz, Frank B. Hu, Angela D. Liese, Francine Laden

**Affiliations:** 1 South Carolina Cancer Prevention and Control Program and Department of Environmental Health Sciences, Arnold School of Public Health, University of South Carolina, Columbia, South Carolina, USA; 2 Department of Epidemiology, Harvard School of Public Health, Boston, Massachusetts, USA; 3 Channing Laboratory, Department of Medicine, Brigham and Women’s Hospital and Harvard Medical School, Boston, Massachusetts, USA; 4 Exposure, Epidemiology, and Risk Program, Department of Environmental Health, Harvard School of Public Health, Boston, Massachusetts, USA; 5 Department of Nutrition, Harvard School of Public Health, Boston, Massachusetts, USA; 6 Department of Epidemiology and Biostatistics and Center for Research in Nutrition and Health Disparities, Arnold School of Public Health, University of South Carolina, Columbia, South Carolina, USA

**Keywords:** air pollution, diabetes, incidence, particulate matter

## Abstract

**Background:**

Although studies have found that diabetes mellitus (DM) modifies the impact of exposures from air pollution on cardiovascular outcomes, information is limited regarding DM as an air pollution-associated outcome.

**Objectives:**

Using two prospective cohorts, the Nurses’ Health Study (NHS) and the Health Professionals Follow-Up Study (HPFS), we investigated the relationship of incident type 2 DM with exposures to particulate matter (PM) <2.5 μm (PM_2.5_), PM <10 μm (PM_10_), and PM between 2.5 and 10 μm in aerodynamic diameter (PM_10–2.5_) in the previous 12 months and the distance to roadways.

**Methods:**

Cases were reported and confirmed through biennial and supplemental questionnaires of diagnosis and treatment information. During follow-up from 1989 to 2002, questionnaires provided information on time-varying covariates and updated addresses. Addresses were geocoded and used to assign air pollution exposures from spatiotemporal statistical models.

**Results:**

Among participants living in metropolitan areas of the northeastern and midwestern United States, there were 3,784 incident cases of DM in the NHS, and 688 cases in the HPFS. Pooled results from random effects meta-analysis of cohort-specific models adjusted for body mass index and other known risk factors produced hazard ratios (HRs) for incident DM with interquartile range (IQR) increases in average PM during the 12 months before diagnosis of 1.03 [95% confidence interval (CI), 0.96–1.10] for PM_2.5_, 1.04 (95% CI, 0.99–1.09) for PM_10_, and 1.04 (95% CI, 0.99–1.09) for PM_10–2.5._ Among women, the fully adjusted HR for living < 50 m versus ≥ 200 m from a roadway was 1.14 (95% CI, 1.03–1.27).

**Conclusions:**

Overall, results did not provide strong evidence of an association between exposure to PM in the previous 12 months and incident DM; however, an association with distance to road (a proxy marker of exposure to traffic-related pollution) was shown among women.

The association between cardiovascular outcomes and exposure to ambient levels of air pollutants is well supported in the literature (Dockery et al. 1993; Laden et al. 2006; Miller et al. 2007; Pope et al. 2002; Puett et al. 2008, 2009). Studies have also reported that individuals with diabetes mellitus (DM) have increased susceptibility for adverse cardiovascular outcomes related to acute increases in exposures to air pollution (Bateson and Schwartz 2004; Dubowsky et al. 2006; O’Neill et al. 2005; Peel et al. 2007; Zanobetti and Schwartz 2001). What is not known is whether DM is in itself an adverse outcome of air pollution.

There are few studies exploring a potential causal role of air pollution in DM development. Brook et al. (2008) studied the relationship between DM and exposures to traffic pollution among more than 7,600 men and women in two Canadian cities using nitrogen dioxide (NO_2_) measurements taken from 2002 to 2004. Meta-analytic models showed a statistically significant increase in the odds of DM among women with each increase in 1-ppb exposure to NO_2_. No association was found among male study participants. A case–control study reported that prediagnosis average particulate matter (PM) < 10 μm in aerodynamic diameter (PM_10_) exposure was significantly higher for children diagnosed with DM compared with controls (Hathout et al. 2002). Finally, diabetes-related mortality has been associated with PM and other ambient air pollutant exposures (Kan et al. 2004; Maynard et al. 2007; Ostro et al. 2006). However, based on studies (Bateson and Schwartz 2004; Dubowsky et al. 2006; O’Neill et al. 2005; Peel et al. 2007; Zanobetti and Schwartz 2001) that showed DM as an effect modifier of air pollution–associated DM outcomes, these findings may reflect susceptibility rather than incidence.

In this study, we used data from two established prospective cohorts, the Nurses’ Health Study (NHS) and the Health Professionals Follow-up Study (HPFS), to examine the role of chronic exposures to PM < 2.5 μm in aerodynamic diameter (PM_2.5_), PM_10_, PM between 2.5 and 10 μm in aerodynamic diameter (PM_10–2.5_), and proximity to roads (as a proxy for traffic-related pollutant exposures) as risk factors for type 2 DM. Biennial questionnaires from each of these cohorts and detailed monthly exposure modeling provided a unique opportunity to control for known individual-level risk factors and to estimate ambient PM exposures specific to all reported mailing addresses for each participant during the 13-year follow-up period.

## Materials and Methods

### Study population

The NHS is a prospective cohort study that began in 1976 with 121,700 female registered nurses age 30–55 years who lived in one of 11 states in the United States (California, Texas, Florida, Massachusetts, Pennsylvania, Ohio, New York, New Jersey, Michigan, Connecticut, and Maryland) at enrollment. Biennial questionnaires on risk factors and health outcomes have been mailed to participants from 1976 until the present (NHS 2009). The NHS was approved by the institutional review board (IRB) of the Brigham and Women’s Hospital.

The HPFS cohort originated in 1986 with 51,529 male dentists, podiatrists, pharmacists, veterinarians, osteopaths, and optometrists located throughout the United States who were 40–75 years of age at enrollment. In 1986, the participants returned a mailed questionnaire that collected data on lifestyle characteristics and medical history. As with the NHS, HPFS participants also were mailed questionnaires every 2 years to date (HPFS 2009). The HPFS was approved by the Harvard School of Public Health IRB. For both of these cohorts, baseline IRB approval included returning the completed questionnaires, which constituted implied consent to use the data in ongoing health research. Loss to follow-up in both of these cohorts is < 10%.

This study was restricted to NHS and HPFS participants living in metropolitan statistical areas (MSAs) in 13 U.S. contiguous northeastern and midwestern states (Maine, Vermont, New Hampshire, Massachusetts, Rhode Island, Connecticut, New York, New Jersey, Delaware, Pennsylvania, Ohio, Michigan, and Maryland) from 1989 through 2002 to facilitate comparisons with previous studies (Eftim et al. 2008; Pope et al. 1995, 2002) and because air pollution monitors used to estimate exposures that are sparsely distributed outside the MSAs. In addition, we excluded persons with a history of diabetes at baseline. The final study population comprised 74,412 women and 15,048 men, with the vast majority of exclusions from the original cohort population attributable to addresses outside the Northeast and the Midwest at baseline, rather than addresses outside an MSA. Nurses and health professionals were excluded for any period of follow-up during which they lived outside this region, rather than being censored at the time they moved outside the region.

### Outcome assessment

Study participants who reported a diagnosis of DM on a biennial questionnaire were sent an additional questionnaire to ascertain the month and year of diagnosis and information about diagnostic tests and treatment. To be considered a confirmed case of DM, at least one of the following National Diabetes Data Group criteria (National Diabetes Data Group 1979) had to be met: elevated plasma glucose concentrations on at least two different occasions (as defined below), one or more DM symptoms (e.g., weight loss, thirst, polyuria) and a single elevated plasma glucose concentration, or treatment with hypoglycemic medication. An elevated plasma glucose concentration was defined as a fasting plasma glucose > 140 mg/dL for cases diagnosed before or during 1997 or > 126 mg/dL for cases diagnosed after 1997 (Expert Committee on the Diagnosis and Classification of Diabetes Mellitus 1997), a random plasma glucose concentration > 200 mg/dL, or a plasma glucose concentration > 200 mg/dL after > 2 hr of oral glucose tolerance testing. Comparisons between medical records and self-reported DM for subsamples of men from the HPFS cohort and women from the NHS resulted in confirmation of 97% and 98% of cases, respectively (Hu et al. 2001a; Manson et al. 1991).

### Exposure assessment

To ascertain the exposure of each participant to air pollution at each geocoded address where questionnaires were mailed, we developed separate spatiotemporal models to estimate monthly PM_2.5_ and PM_10–2.5_ exposures. Mailing addresses were residential for the women; however, some work addresses were included as mailing addresses for the men. These models and their previous use in assessing chronic PM exposures among the NHS cohort are described in detail elsewhere (Paciorek et al. 2009; Puett et al. 2008, 2009; Yanosky et al. 2008, 2009). Briefly, a PM_10_ model was developed first, using monitor data from the U.S. Environmental Protection Agency (EPA) Air Quality System (AQS; U.S. EPA 2009), the Visibility Information Exchange Web System (VIEWS 2004), the Interagency Monitoring of Protected Visual Environments (IMPROVE) network, Stacked Filter Unit (a predecessor to IMPROVE), Clean Air Status and Trends (CASTNet) networks, and Harvard research studies such as the Twenty-four Cities Study and Five Cities Study (Spengler et al. 1996; Suh et al. 1997). The model also included geographic information system (GIS)–derived covariates such as meteorology, land use and elevation, population density, road network, and point source emission data. Land use data were from the U.S. Geological Survey 1992 National Land Cover Data Set (Yanosky et al. 2008). The process of estimating PM_2.5_ exposures was similar to the process used to estimate PM_10_ exposures but involved developing separate models for PM_2.5,_ before and after 1999, because U.S. EPA AQS monitoring data for PM_2.5_ were unavailable before 1999. The pre-1999 PM_2.5_ model used a simpler spatiotemporal structure to estimate the PM_2.5_ to PM_10_ ratio seasonally and included estimated extinction coefficients from airport visibility data. Finally, PM_10–2.5_ exposures were estimated, for each month and location, by subtracting each of the modeled PM_2.5_ estimates from each of the modeled PM_10_ estimates. Monitor data were included from 922 PM_10_ sites and 498 PM_2.5_ sites (Paciorek et al. 2009). The PM_10_ model and post-1999 and pre-1999 PM_2.5_ models were evaluated using a cross-validation approach, where a subselection of monitors were held out to compare predicted values with observed values (Paciorek et al. 2009; Yanosky et al. 2008, 2009). The models were shown to exhibit little bias and high precision. For the PM_10_ model, cross-validation (*R*^2^ = 0.62) showed the model performed substantially better than other approaches (e.g., inverse distance weighting cross-validation *R*^2^ = 0.29) (Yanosky et al. 2008). Cross-validation for the PM_2.5_ model was *R*^2^ = 0.77 and 0.69 for post-1999 and pre-1999 PM_2.5_ models, respectively (Yanosky et al. 2009). The predicted PM_10–2.5_ levels showed little bias but were less precise compared with PM_2.5_ (Yanosky et al. 2009). Yanosky et al. (2009) noted that the PM_2.5_ levels in the study area were more spatially homogenous than were the PM_10_ and PM_10–2.5_ levels.

Distance from each biennially updated address to the nearest road was used as a proxy for traffic-related air pollution exposure. Specifically, the distance (in meters) from each address to the closest U.S. census feature class code A1 (roads with limited access, typically interstates), A2 (major, noninterstate roads), or A3 (secondary roads, typically with more than two lanes) road segment was determined using a GIS (ArcGIS, version 9.2; ESRI, Redlands, CA) and 2000 U.S. Census Topologically Integrated Geographic Encoding and Referencing system (TIGER) files (U.S. Census 2000). Based on information from previous studies and the exposure distributions in these cohorts, distance to the closest road was categorized as 0–49 m, 50–99 m, 100–199 m, or ≥ 200 m (Adar and Kaufman 2007; Hart et al. 2009; Lipfert and Wyzga 2008; Zhu et al. 2002).

### Covariates

To assess potential confounding and effect modification, time-varying data from the biennial questionnaires were used for the following covariates: hypertension (yes or no regarding a diagnosis from a health professional), smoking status (never, former, or current), hypercholesterolemia (yes or no regarding a diagnosis from a health professional), alcohol consumption (0, 0.1–4.9, 5.0–14.9, ≥15 g/day), and smoking pack-years. Having a low-risk diet (yes, no) was assessed by semiquantitative food frequency questionnaires administered every 4 years and defined as a diet with a high ratio of polyunsaturated to saturated fat that also was high in cereal fiber and low in trans fat and glycemic load (details described elsewhere) (Hu et al. 2001b). Baseline body mass index (BMI; < 25.0, < 30.0, or ≥ 30.0 kg/m^2^) and physical activity [< 3, 3 to < 9, 9 to < 18, 18 to < 27, or ≥27 metabolic equivalent (MET) hr/week] were also included in models. We modeled baseline rather than time-varying values of BMI and physical activity to avoid adjusting for factors that might be a consequence of the outcome, given that insulin resistance and DM could lead to reduced physical activity and thus increased BMI. For selection of covariates in final fully adjusted models, we evaluated changes (10% difference) in model estimates, but also opted to include some variables that might not have produced such a change in estimates but that are known risk factors for diabetes based on prior knowledge. Although some of the covariates may not have been identified previously as predictors of the exposure, it can be useful to adjust for these factors, as they reduce unexplained variation in the outcome and therefore increase power to detect the exposure.

### Statistical analysis

Time-varying Cox proportional hazards models were used to assess the relationship of DM with predicted PM_2.5,_ PM_10_, and PM_10–2.5_ exposures in the 12 months before diagnosis and traffic-related exposures, with each cohort analyzed individually. All survival models were based on a monthly time scale and were used to estimate hazard ratios (HRs) and 95% confidence intervals (CIs). We assessed PM exposure averaged over the 12 months before DM diagnosis because longer windows of exposure were highly correlated, and previous research has shown that time period to be the most relevant exposure for air pollution-related deaths (Schwartz et al. 2008). Person-months of follow-up time were calculated from baseline (30 June 1989 for NHS and 30 January 1989 for HPFS) until DM diagnosis, the end of follow-up (30 June 2002 for NHS and 30 January 2002 for HPFS), censoring (moving outside the geographic region of interest or loss to follow-up), or death. Separate models assessed each particulate fraction alone, and a combined model examined PM_2.5_ and PM_10–2.5_ simultaneously. Cox models were stratified by age in months and adjusted for year (linear term) and state of residence. Because our PM analyses focused on exposures averaged over 12 months, we also stratified by season to adjust for seasonal changes in PM. Person-time spent living outside the geographic region of interest was excluded, rather than censoring the participant at the time of the move, as were nurses and health professionals with DM reported before baseline. Confounders were included in multivariable models individually, and HR modification was evaluated using *p*-values (< 0.05) from multiplicative interaction terms. In sensitivity analyses, we conducted analyses excluding women and men who reported myocardial infarctions (MIs) and cancer (except nonmelanoma skin cancer) prior to baseline. In addition, we performed sensitivity analyses restricted to symptomatic cases, defined as cases who had at least one of the following symptoms at diagnosis: ketoacidosis, unusual urinary frequency, coma, unusual hunger, unintended weight loss, unusual thirst, and visual changes for health professionals and nurses and, additionally, pruritis of the vulva/vagina for nurses. To explore potential changes in the relationship of DM to air pollution exposures over time, we additionally estimated associations between DM and air pollution exposures during the first 2 years of follow-up and during the previous 2 years of follow-up, as well as for the average exposure across the study period. Random effects models were used to conduct a pooled meta-analysis to increase precision of the risk estimates and CIs, and heterogeneity was evaluated with the Q test (DerSimonian and Laird 1986). We performed all the statistical analyses using SAS (version 9.1; SAS Institute Inc., Cary, NC).

## Results

There were 3,784 incident cases of DM among 74,412 eligible participants in the NHS (448 per 100,000 person-years) and 688 cases among 15,048 eligible participants in the HPFS (402 per 100,000 person-years) (Table 1). At baseline in 1989, the mean age was approximately 57 years for HPFS and 55 years for NHS participants. Most were never (NHS: 43%, HPFS: 45%) or former smokers (NHS: 36%, HPFS 46%). NHS participants were more likely than HPFS participants to have baseline BMI < 25 or > 30, although the prevalence of hypertension at baseline was similar for both groups. Men in the HPFS were more physically active and consumed more alcohol than did women in the NHS. The means and SDs of baseline particulate exposures for the HPFS and NHS were similar for PM_2.5_ [18.3 (3.1) and 17.5 (2.7) μg/m^3^], PM_10_ [28.5 (5.5) and 26.9 (4.8) μg/m^3^], and PM_10–2.5_ [10.3 (3.3) and 9.4 (2.9) μg/m^3^]. Baseline addresses for HPFS participants were more likely than NHS addresses to be 0–49 m from the nearest road (22.3% vs. 9.8%) and less likely to be ≥ 200 m from the nearest road (63.7% vs. 77.5%).

Among the NHS, an interquartile range (IQR) increase of 4 μg/m^3^ in estimated PM_10–2.5_ averaged over the 12 months prior to diagnosis was associated with incident DM (HR = 1.07; 95% CI, 1.01–1.13) based on a single pollutant model stratified by age and adjusted for state of residence, year, and season (Table 2), but the HR was attenuated after additional adjustment for cigarette smoking, hypertension, BMI, alcohol intake, physical activity and diet (HR = 1.04; 95% CI, 0.98–1.10). Associations with IQR increases in PM_2.5_ (IQR: 4 μg/m^3^) and PM_10_ (IQR: 6 μg/m^3^) were similar to those estimated for PM_10–2.5_ in fully adjusted models (PM_2.5_ HR = 1.02; 95% CI, 0.94–1.09; PM_10_ HR = 1.03; 95% CI, 0.98–1.09), and associations with PM_2.5_ and PM_10–2.5_ were similar when both were included in the same model. Estimates did not change appreciably when time-varying rather than baseline BMI was included or when family history of diabetes, census tract median household value, or census tract median household income were added to models (data not shown). NHS participants living < 50 m from the nearest road were more likely to be diagnosed with DM than those living ≥ 200 m away (fully adjusted HR = 1.14; 95% CI, 1.03–1.27) (Table 3). A nonsignificant (*p* > 0.05) association was also evident for women with residences located 50–100 m from the nearest road.

Among the HPFS, estimated associations with DM were similar for IQR increases in PM_2.5,_ (IQR: 4 μg/m^3^), PM_10,_ (IQR: 7 μg/m^3^), and PM_10–2.5_ (IQR: 4 μg/m^3^) averaged over the previous 12 months (fully adjusted HR = 1.07; 95% CI, 0.92–1.24; 1.06, 95% CI, 0.94–1.20; and 1.04, 95% CI, 0.93–1.16, respectively) (Table 2). Estimated associations were similar when measures of neighborhood socioeconomic status (SES), time-varying rather than baseline BMI, and family history of diabetes were added to fully adjusted models (data not shown). Unlike the findings among women in the NHS with the fully adjusted multipollutant model, the risk of DM was greater for PM_2.5_ (HR = 1.06; 95% CI, 0.89–1.26) than PM_10–2.5_ (HR = 1.02; 95% CI, 0.89–1.16). In contrast with findings for the NHS, distance to the nearest road was not associated with incident DM among men in the HPFS (Table 3).

Pooled meta-analysis models adjusted for age, year, season, and state suggested a slight increase in diabetes incidence associated with an IQR increase in estimated PM_10–2.5_ and PM_10_ averaged over the 12 months prior to diagnosis (for both exposures: HR = 1.06; 95% CI, 1.01–1.12), although associations were attenuated after full adjustment (for both exposures: HR = 1.04; 95% CI, 0.99–1.09) (Table 2). Associations with PM_2.5_ were somewhat weaker. Tests of heterogeneity between the NHS and HPFS were not significant for any of the pooled meta-analysis models, but a multiplicative interaction term between sex and PM_2.5_ was statistically significant in a fully adjusted model (*p* = 0.04) (data not shown). The pooled meta-analysis of DM incidence and proximity to roadways indicated a statistically significant association with an address 0–49 m versus ≥ 200 m from the nearest road (HR = 1.11; 95% CI, 1.01–1.23) (Table 3).

Estimates from models adjusted for age, year, season, and state of residence, plus individual covariates were generally similar to estimates from fully adjusted models (data not shown). However, relative to basic models, adjusting for baseline BMI slightly attenuated associations with all size fractions of PM in the NHS (e.g., HR for PM_2.5_ in basic model adjusting for BMI = 1.02; 95% CI, 0.94–1.09) but slightly increased the HR for PM_2.5_ in the HPFS (HR = 1.08; 95% CI, 0.93–1.25). No evidence of effect modification was found for any of the covariates examined (data not shown).

Estimates from models that excluded participants with a baseline history of MI (leaving 3,672 NHS and 604 HPFS cases) or prior cancer (except nonmelanoma skin cancer) (leaving 3,311NHS and 522 HPFS cases) were similar to the reported estimates (data not shown). Compared with estimates from our original HPFS study population, analyses restricted to symptomatic DM cases (366) suggested a slightly lower, although still null, risk of incident DM with an IQR increase in PM exposures (fully adjusted PM_2.5_ HR = 0.96; 95% CI, 0.78–1.17; PM_10_ HR = 0.97; 95% CI, 0.82–1.15; PM_10–2.5_ HR = 0.98; 95% CI, 0.85–1.14). Results for symptomatic NHS participants (3,379) were comparable with those for all cases (data not shown).

In sensitivity analyses restricted to the first 2 years of follow-up, relative risks (RRs) for NHS women with IQR increases in all three particle fraction exposures were lower than for the full time period of follow-up, but higher when analyses were restricted to the final 2 years of follow-up (Table 4). Among men in the HPFS, associations were stronger with PM_2.5_ during the first 2 years and with all size fractions in the last 2 years than for the full period of follow-up, whereas the association with PM_10–2.5_ during the first 2 years was weaker.

When associations were estimated with IQR increases in PM exposures averaged over the entire follow-up period (instead of during the 12 months prior to diagnosis), there was no evidence of associations between incident DM and any PM size fraction among NHS women, and associations with PM_2.5_ and PM_10_ were attenuated for HPFS men (data not shown). In pooled meta-analyses, HRs for incident DM associated with average PM exposures over the entire follow-up period were 0.99 (95% CI, 0.89–1.10) for PM_2.5_; 0.98 (95% CI, 0.91–1.06) for PM_10_; and 1.02 (95% CI, 0.93–1.12) for PM_10–2.5_.

## Discussion

Overall findings for these two cohorts of nurses and health professionals living in the Northeast and Midwest indicate weak nonstatistically significant increased risks of incident DM associated with IQR increases in predicted PM_2.5_, PM_10_, and PM_10–2.5_ averaged over the 12 months prior to diagnosis. RR estimates were very similar for each size PM fraction, with overlapping CIs. Among NHS participants, the strongest fully adjusted association was with PM_10–2.5_, whereas the weakest was with PM_2.5._ This pattern was reversed in the HPFS, with the weakest association observed for PM_10–2.5_ and the strongest with PM_2.5_. Although the addition of a multiplicative interaction term in the fully adjusted model for PM_2.5_ was significant, showing a greater risk among men, the pooled meta-analysis did not show a difference, possibly because of differences in how each analytic method deals with adjustment by covariates. Multipollutant models, analyses restricted to symptomatic DM, and analyses restricted to the first 2 years of follow-up were similar. In general, results were weaker for PM exposures averaged over the entire follow-up period. RRs were stronger for DM during the final 2 years of follow-up, although CIs were wider because of the reduction in sample size. Finally, fully adjusted estimates showed a statistically significant association with DM for NHS women living < 50 m versus > 200 m from the nearest road. However, distance to the nearest road was not associated with DM among the HPFS.

The body of research on associations between diabetes and air pollution exposures is currently very limited. Although direct comparisons with our study results are not possible because of differences in the pollutants, diabetes type, and age groups studied, other studies have also shown weak evidence of an association between diabetes and PM exposures and stronger evidence for traffic-related pollutants (represented by distance from the nearest road in our study). Brook et al. (2008) reported a 4% increase in the adjusted odds (95% CI, 1.00–1.08, *p* = 0.03) of DM diagnosis with each ppb increase in NO_2_ exposure among 4,182 women in Hamilton and Toronto, Canada, but no association among the 3,452 men in the study [odds ratio (OR) = 0.99; 95% CI, 0.95–1.03]. In a study of 402 children in Southern California, type 1 diabetes was not significantly associated with a 10-μg/m^3^ increase in average PM_10_ from birth to diagnosis (OR = 1.08; 95% CI, 0.87–1.34) or a 10-ppb increase in NO_2_ (OR = 1.03; 95% CI, 0.71–1.50) but was significantly associated with a 10-ppb increase in O_3_ (OR = 2.92; 95% CI, 1.86–4.58) and SO_4_ (OR = 1.65; 95% CI, 1.20–2.28) (Hathout et al. 2006). In a smaller study of 100 children, designed as two case–control studies—one among younger children and another among older children, Hathout et al. (2002) reported a significant association between type 1 diabetes diagnosis and average prediagnosis PM_10_ among children under age six years, but not among older children. In both age groups, type 1 diabetes was associated with average birth to diagnosis exposure to O_3_, but not to NO_2_ or SO_4_.

Evidence of associations between air pollution exposures and diabetes mortality is also somewhat inconsistent. In a time series study of 434 diabetes deaths in Shanghai, Kan (2004) found a weak association with a 10-μg/m^3^ increase in 1-day lagged PM_10_ (RR = 1.01; 95% CI, 1.00–1.01) and a 10-μg/m^3^ increase in 1-day lagged NO_2_ (RR = 1.01; 95% CI, 1.00–1.03). A study of mortality among Montreal, Canada, residents registered with the universal Quebec Health Insurance Plan included 3,677 deaths from diabetes (Goldberg et al. 2006). Some associations were reported between daily diabetes mortality and the 3-day mean during the warm or cold season for certain air pollutants generated from combustion sources (e.g., sulfate, PM_2.5_, NO_2_) in individuals who also had cardiovascular disease, cancer, or respiratory disease. No associations were reported for individuals without these conditions. In a case-crossover study of 100,000 deaths from 1995 to 2002, an interquartile increase (0.203 g/m black carbon) in traffic particle exposure the day before death was associated with a 5.7% (95% CI, –1.7 to 13.7) increase in deaths due to DM (Maynard et al. 2007).

Biological mechanisms proposed to explain associations between air pollution and cardiovascular disease, specifically, inflammation, oxidative stress, and endothelial dysfunction, are also plausible mechanisms linking air pollution with the development or exacerbation of diabetic conditions. However, the majority of literature to date suggests that DM is an effect modifier of the relationship between air pollution exposures and cardiovascular outcomes, as opposed to being a direct consequence of air pollution exposures. Zanobetti and Schwartz (2001, 2002) reported that the percent increase in cardiovascular hospitalizations associated with a 10-μg/m^3^ increase in mean PM_10_ exposure on the day of and day before admission was almost doubled among persons with diabetes compared with the percent increase among those without diabetes. Comparable findings of stronger associations between air pollution exposures and cardiovascular-associated hospitalizations and emergency department visits among persons with diabetes compared with those without diabetes have been reported by other researchers (Peel et al. 2007; Pereira Filho et al. 2008). Research has suggested that inflammatory mechanisms are involved in strengthening the impact of air pollution among persons with diabetes. Increased levels of inflammatory biomarkers, C-reactive protein and IL-6 were more strongly associated with PM_2.5_ exposures among persons with diabetes compared with persons without the disease (Dubowsky et al. 2006). Decreases in flow-mediated vascular reactivity and nitroglycerin-mediated reactivity were associated with increases in 6-day moving average black carbon and PM_2.5_ exposures, respectively, among a population of diabetics, whereas similar decreases were not evident among those at risk for diabetes (O’Neill et al. 2005).

Although this study helps address the gap of information regarding direct links between air pollution and diabetes, limitations and strengths must be considered. We included only confirmed and probable DM cases based on National Diabetes Group and American Diabetes Association criteria (National Diabetes Data Group 1979); however some persons with diabetes may have been misclassified because we relied initially on self-reported diagnosis rather than on glucose testing for all cohort participants. Although we had the advantage of using data from large prospective cohorts, our power to detect an effect among the HPFS men was somewhat limited by the sample size, particularly in sensitivity analyses restricted to symptomatic DM cases. In addition, the meta-analyses and combined analyses with sex-interaction terms were dominated by the NHS because of a smaller number of HPFS participants. In addition, although results were consistent for the two time periods of average PM exposure, we evaluated (12 months before diagnosis and exposures averaged over the entire follow-up period), additional time windows of exposure should be explored, including more acute exposures and exposures during childhood.

As in our previously published studies of the NHS (Puett et al. 2008, 2009), the GIS-based temporal spatial smoothing of our PM predictive models reduced variability relative to measured concentrations. This Berkson measurement error should not cause substantial bias toward the null (Gryparis et al. 2009). However, it is possible that differences between study populations with respect to traffic-related exposures (distance to the nearest road) and PM_2.5_ associations, may be due in part to differences in the type of address available for each study—residential only in the NHS versus occupational or residential in the HPFS—rather than differences in susceptibility between men and women. Thus, error in the predicted exposure estimates for the HPFS would differ from the NHS. For the HPFS, however, both address types are likely to represent PM exposures during a large part of a 24-hr period, albeit different parts. Finally, unlike many previous studies, the availability of geocoded biennially updated addresses enabled us to adjust for a number of biennially updated individual-level covariates and to assign predicted particulate exposure levels to each address for each participant throughout the follow-up period. Therefore, exposure estimates should be more accurate over time than estimates based on baseline addresses only. In addition, missing exposure data were minimized because of the use of a recently developed and highly specific GIS-based spatial smoothing model.

Because of the mixed nature of the HPFS addresses, we did not believe it was appropriate to assess area-level SES as a confounder in this study. However, estimates from models adjusted for neighborhood-level SES, such as median house value and average household income at the census-tract level (U.S. Census 2000) were similar to those presented for both the HPFS and NHS. An additional potential limitation of our analysis was that our two populations of health professionals represent a narrow range of SES, possibly limiting the generalizability of our results.

## Conclusion

In summary, we did not find strong evidence for an association between exposure to PM_2.5_, PM_10_, or PM_10–2.5_ in the 12 months before diagnosis and type 2 DM incidence among female nurses and male health professionals living in the northeastern and midwestern United States. However, our findings did suggest an association between residential proximity to roadways and incident DM among female nurses. Although questions remain regarding which pollutants play the most prominent role in incident DM and which subpopulations are most susceptible, our study results add to other findings reported in the current literature suggesting that DM may be an outcome of air pollution exposures in addition to being an effect modifier for air pollution–associated cardiovascular disease.

## Figures and Tables

**Table 1:** Descriptive characteristics of the NHS and HPFS participants at baseline, 1989.

Characteristic	HPFS	NHS
Participants (*n*)	15,048	74,412
Cases (*n*)	688	3,784
Age (years)	57.3 ± 9.7	55.1 ± 7.1
< 25.0	45.4	53.1
< 30.0	46.0	30.6
> 30.0	8.5	16.3
Smoking status		
Never	44.9	43.0
Current	9.4	20.7
Former	45.7	36.3
Pack-years of smoking	25.0 ± 19.4	23.7 ± 19.4
Hypertension	26.1	26.8
High-risk diet	56.6	61.8
Alcohol consumption (g/day)		
0	20.5	34.3
0.1–4.9	28.2	34.1
5.0–14.9	28.0	20.5
≥ 15.0	23.3	11.0
Physical activity (MET/hr/week)		
< 3	15.2	25.3
3 to < 9	19.0	27.7
9 to < 18	19.0	20.0
18 to < 27	13.9	11.3
≥ 27	32.9	15.7
Predicted PM_2.5_ (μg/m^3^)^a^	18.3 ± 3.1	17.5 ± 2.7
IQR predicted PM_2.5_ (g/m^3^)^a^	4.0	4.3
Predicted PM_10–2.5_ (μg/m^3^)^a^	10.3 ± 3.3	9.4 ± 2.9
IQR predicted PM_10–2.5_ (μg/m^3^)^a^	4.2	3.7
Predicted PM_10_ (μg/m^3^)^a^	28.5 ± 5.5	26.9 ± 4.8
IQR predicted PM_10_ (μg/m^3^)^a^	7.2	6.3
Distance to road (m)		
0–49	22.3	9.8
50–99	4.4	3.8
100–199	9.6	8.9
≥ 200	63.7	77.5

Data are presented as mean ± SD or percentages.

a For 12 months prior.

**Table 2:** HRs (95% CIs) for diabetes associated with an IQR increase^a^ in average-predicted PM exposure in the 12 months before diagnosis.

	NHS (3,784 cases, 844,490 person-years)		HPFS (688 cases, 167,310 person-years)		Pooled meta-analysis	
	Age, season, year, state adjusted	Fully adjusted^b^	Age, season, year, state adjusted	Fully adjusted^b^	Age, season, year, state adjusted	Fully adjusted^b^
Single pollutant models						

PM_2.5_	1.04 (0.97–1.12)	1.02 (0.94–1.09)	1.05 (0.91–1.22)	1.07 (0.92–1.24)	1.05 (0.98–1.12)	1.03 (0.96–1.10)
PM_10_	1.06 (1.00–1.12)	1.03 (0.98–1.09)	1.06 (0.93–1.20)	1.06 (0.94–1.20)	1.06 (1.01–1.12)*	1.04 (0.99–1.09)
PM_10–2.5_	1.07 (1.01–1.13)*	1.04 (0.98–1.10)	1.05 (0.94–1.17)	1.04 (0.93–1.16)	1.06 (1.01–1.12)*	1.04 (0.99–1.09)

Multipollutant models						

PM_2.5_	1.01 (0.93–1.09)	0.99 (0.92–1.08)	1.04 (0.91–1.18)	1.06 (0.89–1.26)	1.01 (0.94–1.09)	1.00 (0.93–1.08)
PM_10–2.5_	1.06 (1.00–1.13)	1.04 (0.98–1.11)	1.02 (0.86–1.22)	1.02 (0.89–1.16)	1.06 (1.00–1.12)*	1.04 (0.98–1.10)

a PM_2.5_ IQR = 4 μg/m^3^; PM_10,_ IQR = 7 μg/m^3^; and PM_10–2.5_ IQR = 4 μg/m^3^.

b Adjusted for age, season, calendar year, state of residence, time-varying cigarette smoking (status and pack-years), time-varying hypertension, baseline BMI, time-varying alcohol intake, baseline physical activity, and time-varying diet.

* *p* < 0.05.

**Table 3:** HRs and 95% CIs for diabetes associated with proximity to nearest road.

	NHS (4,037 cases, 869,072 person-years)		HPFS (715 cases, 172,283 person-years)		Pooled meta-analysis	
	Age, season, year, state adjusted	Fully adjusted^a^	Age, season, year, state adjusted	Fully adjusted^a^	Age, season, year, state adjusted	Fully adjusted^a^
Distance to road (m)						

0–49	1.20 (1.08–1.33)*	1.14 (1.03–1.27)*	0.99 (0.82–1.19)	1.02 (0.85–1.23)	1.11 (0.92–1.33)	1.11 (1.01–1.23)
50–99	1.20 (1.03–1.40)*	1.16 (0.99–1.35)	0.76 (0.51–1.14)	0.74 (0.49–1.11)	0.99 (0.64–1.54)**	0.96 (0.63–1.48)**
100–199	1.02 (0.92–1.14)	0.97 (0.88–1.08)	0.86 (0.66–1.13)	0.88 (0.67–1.16)	0.99 (0.86–1.13)	0.96 (0.87–1.06)
≥ 200	1.00 (referent)	1.00	1.00	1.00	1.00	1.00

a Adjusted for age, season, calendar year, state of residence, time-varying cigarette smoking (status and pack-years), time-varying hypertension, baseline BMI, time-varying alcohol intake, baseline physical activity, and time-varying diet.

* *p* < 0.05.

** *p* < 0.05 for heterogeneity between the NHS and HPFS.

**Table 4:** HRs for diabetes associated with an IQR increase^a^ before 12 months PM exposure for first and final 2 years of follow-up.

	NHS	HPFS
	Fully adjusted model^b^ HR (95% CI)	Fully adjusted model^b^ HR (95% CI)
First 2 years^c^	438 cases, 151,967 person-years	94 cases, 1,996 person-years

PM_2.5_	0.98 (0.80–1.18)	1.13 (0.79–1.62)
PM_10_	1.01 (0.87–1.17)	1.04 (0.76– 1.42)
PM_10–2.5_	1.03 (0.89–1.19)	0.94 (0.70– 1.27)

Last 2 years^d^	593 cases, 135,213 person-years	129 cases, 23,670 person-years

PM_2.5_	1.21 (1.00–1.46)	1.52 (0.93–2.47)
PM_10_	1.13 (0.98–1.29)	1.27 (0.91–1.77)
PM_10–2.5_	1.09 (0.94–1.25)	1.13 (0.88–1.45)

a PM_2.5_ IQR = 4 μg/m^3^; PM_10_ IQR = 7 μg/m^3^; and PM_10–2.5_ IQR = 4 μg/m^3^.

b Adjusted for age, season, calendar year, state of residence, time-varying cigarette smoking (status and pack-years), time-varying hypertension, baseline BMI, time-varying alcohol intake, baseline physical activity, and time-varying diet.

c 1989–1991.

d 2000–2002.
